# *In vivo* redox metabolic imaging of mitochondria assesses disease progression in non-alcoholic steatohepatitis

**DOI:** 10.1038/s41598-017-17447-2

**Published:** 2017-12-07

**Authors:** Ryosuke Nakata, Fuminori Hyodo, Masaharu Murata, Hinako Eto, Tomoko Nakaji, Takahito Kawano, Sayoko Narahara, Keiji Yasukawa, Tomohiko Akahoshi, Morimasa Tomikawa, Makoto Hashizume

**Affiliations:** 1 Department of Advanced Medical Initiatives, Faculty of Medical Sciences, Kyushu University, 3-1-1 Maidashi, Higashi-ku, Fukuoka 812-8582 Japan; 2 Department of Frontier Science for Imaging, School of Medicine, Gifu University, 1-1 Yanagido, Gifu, 501-1194 Japan; 3 Innovation Center for Medical Redox Navigation, Kyushu University, 3-1-1 Maidashi, Higashi-ku, Fukuoka 812-8582 Japan; 4 Center for Advanced Medical Innovation, Kyushu University, 3-1-1 Maidashi, Higashi-ku, Fukuoka 812-8582 Japan; 5 Laboratory of Advanced Pharmacology, Daiichi University of Pharmacy, 22-1 Tamagawa-machi, Minami-ku, Fukuoka 815-0037 Japan

## Abstract

Given the rising incidence of non-alcoholic fatty liver disease (NAFLD) in both adults and children, the development of a non-invasive diagnostic method for assessing disease progression to non-alcoholic steatohepatitis (NASH) has become an important research goal. Currently available non-invasive imaging technologies are only able to assess fat accumulation in the liver. Therefore, these methods are not suitable for a precise diagnosis of NASH. The standard diagnostic technique for NASH, liver biopsy, has several drawbacks, including the higher risk of complications that accompanies invasive procedures. Here, we demonstrated that *in vivo* mitochondrial redox metabolism was dramatically altered at an early stage, before histopathological changes, and NASH could be accurately diagnosed by *in vivo* dynamic nuclear polarization-magnetic resonance imaging, with carbamoyl-PROXYL as a molecular imaging probe. In addition, this technique was feasible for the diagnosis of NASH compared with histopathological findings from biopsies. Our data reveal a novel method for monitoring the dynamics of redox metabolic changes in NAFLD/NASH.

## Introduction

As a consequence of the global epidemic of metabolic syndrome and chronic obesity, non-alcoholic fatty liver disease (NAFLD) has emerged as an important contribution in several chronic liver diseases^1–3^. NAFLD covers a spectrum of histopathological findings, from uncomplicated liver steatosis to non-alcoholic steatohepatitis (NASH). Although NAFLD usually manifests as benign liver steatosis, progression to NASH occurs in approximately 20% of cases. NASH leads to fibrosis in 30 to 40% of patients, and cirrhosis in 10 to 15% of patients, which may ultimately result in hepatocellular carcinoma^4^. Therefore, a precise diagnostic method to distinguish NASH from NAFLD is urgently required. Liver biopsy is the gold standard for diagnosing and staging NASH^5,6^. However, it is an invasive procedure that may cause bleeding or infectious complications, with inspection costs, sampling errors^1,7^, and differences in diagnostic classification among pathologists^8,9^.

Oxidative stress is thought to play a central role in the progression of NAFLD to NASH^3,10–12^. Mitochondria are involved in both ROS generation, and free fatty acid (FFA) ß-oxidation that promotes lipid accumulation. Mitochondrial impairment enhances ROS production, initiating a self-sustaining loop that causes chronic organelle damage. Lipid peroxidation products such as malondialdehyde and 4-hydroxynonenal (4-HNE) inhibit cytochrome c oxidase in mitochondrial complex IV, and ROS may damage mitochondrial DNA (mtDNA) and iron-sulphur cluster enzymes^13^. Indeed, ultrastructural abnormalities and mitochondrial dysfunction are observed in the liver of animal models and patients with NASH^14,15^. Based on these findings, the redox status in NASH, in the context of mitochondria as the main source of ROS, has been investigated^16–18^. However, the role of mitochondrial redox status in the NASH liver is unclear due to a lack of techniques for *in vivo* evaluation, and no reliable diagnostic evaluation is available.

*In vivo* dynamic nuclear polarization-magnetic resonance imaging (DNP-MRI) enables the anatomical distribution of free radical species to be monitored^19,20^. DNP enhances the MRI signal from nuclei such as ^1^H by irradiating tissue at the electron paramagnetic resonance (EPR) frequency of the free radical, before applying the MRI pulse sequence, thus enhancement of the image intensity where free radicals are present^21^. Low-molecular-weight, stable nitroxyl radicals, including carbamoyl-PROXYL (CmP), have been used as probes in a variety of biophysical and biochemical experiments^22^. Nitroxyl compounds including CmP are stable organic free radicals and possessed SOD-like activity. Previous studies revealed that these compounds protected mammalian cells against oxidative damages, despite having no direct catalase activity^23–25^. These results suggested that nitroxyl compounds intercept intracellular oxygen radicals and could exert antioxidant effects. Nitroxyl radicals exist as redox pairs that comprise the free radical form, and the non-paramagnetic hydroxylamine, which is the one-electron reduction product of the nitroxyl radical^26-28^. The reduction rate of the radical probe depends on the tissue redox environment, which is influenced by factors such as overproduction of ROS and reduced antioxidant defences^25,29^. *In vivo* DNP-MRI with nitroxyl radicals has been used to obtain unique information on redox status^30–32^, tissue oxygen partial pressure^33,34^, pH^35^ and proteolytic activity^36^. Studies conducted in the 1970s and 1980s demonstrated that the redox reaction in liver mitochondrial suspensions is modulated by nitroxyl radicals, and reduction of nitroxyl radicals on the mitochondrial membrane is mediated by semiquinones^37^ or the reduced form of coenzyme Q^38^. Here, we evaluated the feasibility of *in vivo* mitochondrial redox status, assessed by DNP-MRI with CmP as a molecular imaging probe, for determining the disease stage in NASH, and to distinguish between NAFLD and NASH.

## Results

The feasibility of *in vivo* DNP-MRI for monitoring the development of NASH was assessed in two types of diet-induced mouse model: a methionine-choline-deficient (MCD) diet and a high-fat (HF) diet. The MCD diet is one of the most commonly used NASH models because mice exhibit severe oxidative stress and hepatocellular injury with fibrosis and inflammation^10,39^. In general, the degree of liver injury is less severe in the HF diet model compared with the MCD diet model^10^. The HF diet requires a longer feeding duration than the MCD diet for mice to display the pathological features of NASH. The use of molecular redox status on *in vivo* DNP-MRI was evaluated as a marker of progression to NASH.

### Liver histopathology and plasma biochemistry

**Figure 1:**
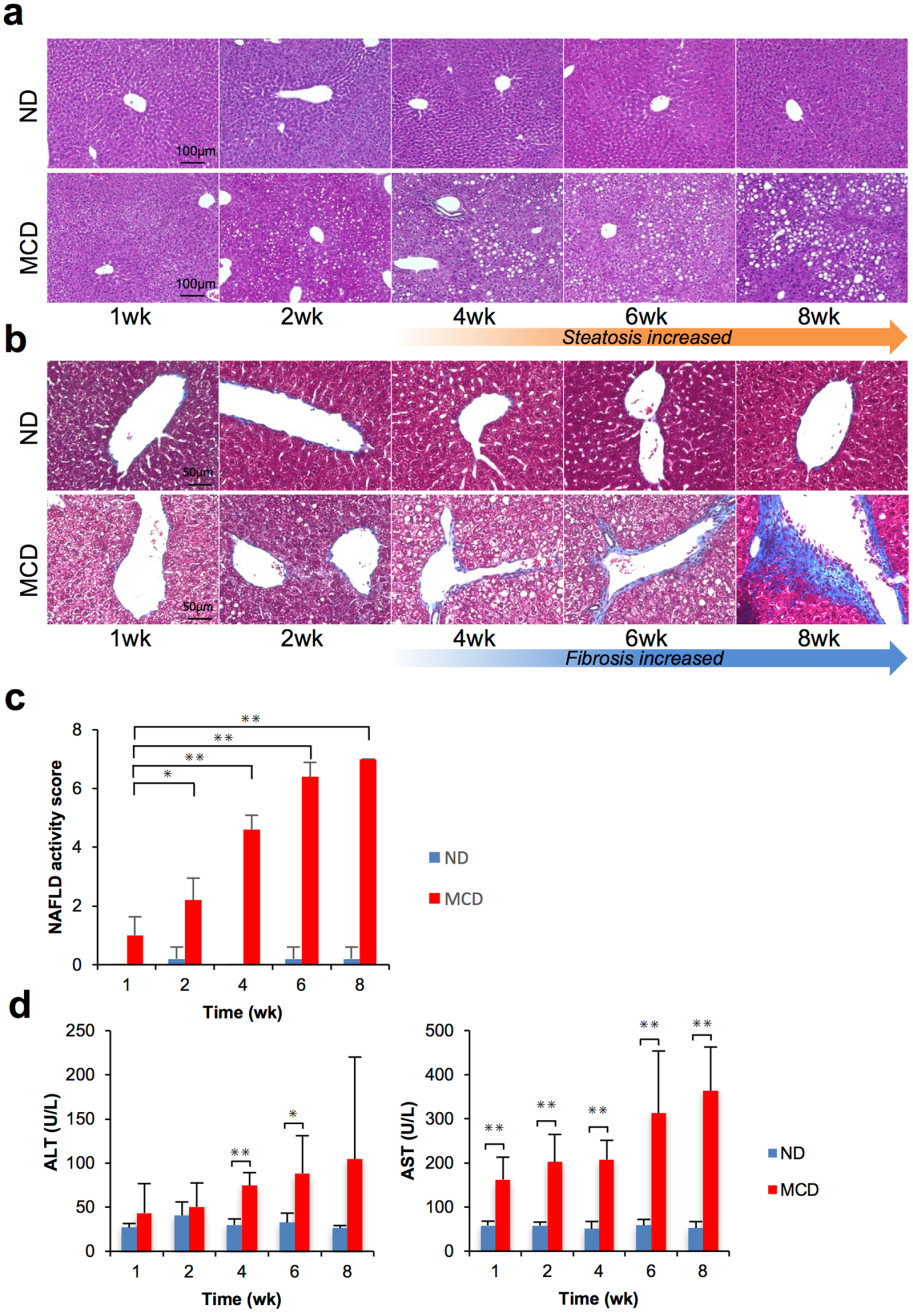
Pathological analysis and assessment of mouse liver tissues after dietary treatment (**a**) Histopathological findings with haematoxylin and eosin (H&E) staining. (**b**) Fibrosis assessment with Masson’s trichrome staining. (**c**) NAFLD activity score. The scores represent the mean ± SD. *p < 0.05, **p < 0.01. (**d**) Plasma biochemistry of alanine transaminase (ALT) and aspartate transaminase (AST) after 1, 2, 4, 6 and 8 weeks. Results represent the mean ± SD (n = 5 per group). ^✳^p < 0.05, ^✳✳^p < 0.01.

All MCD mice had obvious hepatic steatosis, which gradually worsened, depending on the feeding period (Fig. 1a). Hepatic vein fibrosis was observed after 4 weeks of feeding, and fibrotic regions started to develop steatosis (Fig. 1b). Assessment of histopathology with NAFLD activity score (NAS) system presented that score gradually increased in MCD mice (Fig. 1c). NAS is the sum of separate scores for steatosis (0–3), hepatocellular ballooning (0–2), and lobular inflammation (0–3) (Supplementary Fig. S1). Plasma alanine transaminase (ALT) and aspartate transaminase (AST) levels were significantly higher in MCD mice compared with control mice (Fig. 1d).

### Assessment of oxidative stress in MCD mice

**Figure 2:**
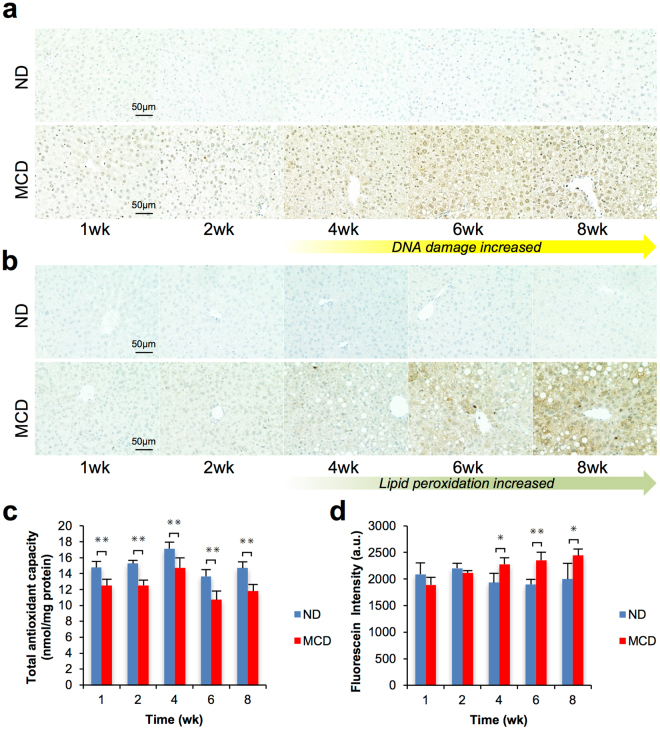
Oxidative stress assessment. (**a**) Immunohistochemistry of 8-OHdG for assessment of oxidized nuclei. (**b**) Immunohistochemistry of 4-hydroxynonenal (4-HNE) for assessment of lipid peroxidation. (**c**) Total antioxidant capacity (TAC) is measured by the reduction reaction of copper ions after 1, 2, 4, 6 and 8 weeks. ^✳✳^p < 0.01. (**d**) ROS production by mitochondria was measured with a MitoSOX-red commercial kit (Thermo Fisher Scientific Inc.) after 1, 2, 4, 6 and 8 weeks. ^✳^p < 0.05, ^✳✳^p < 0.01.

**Figure 4:**
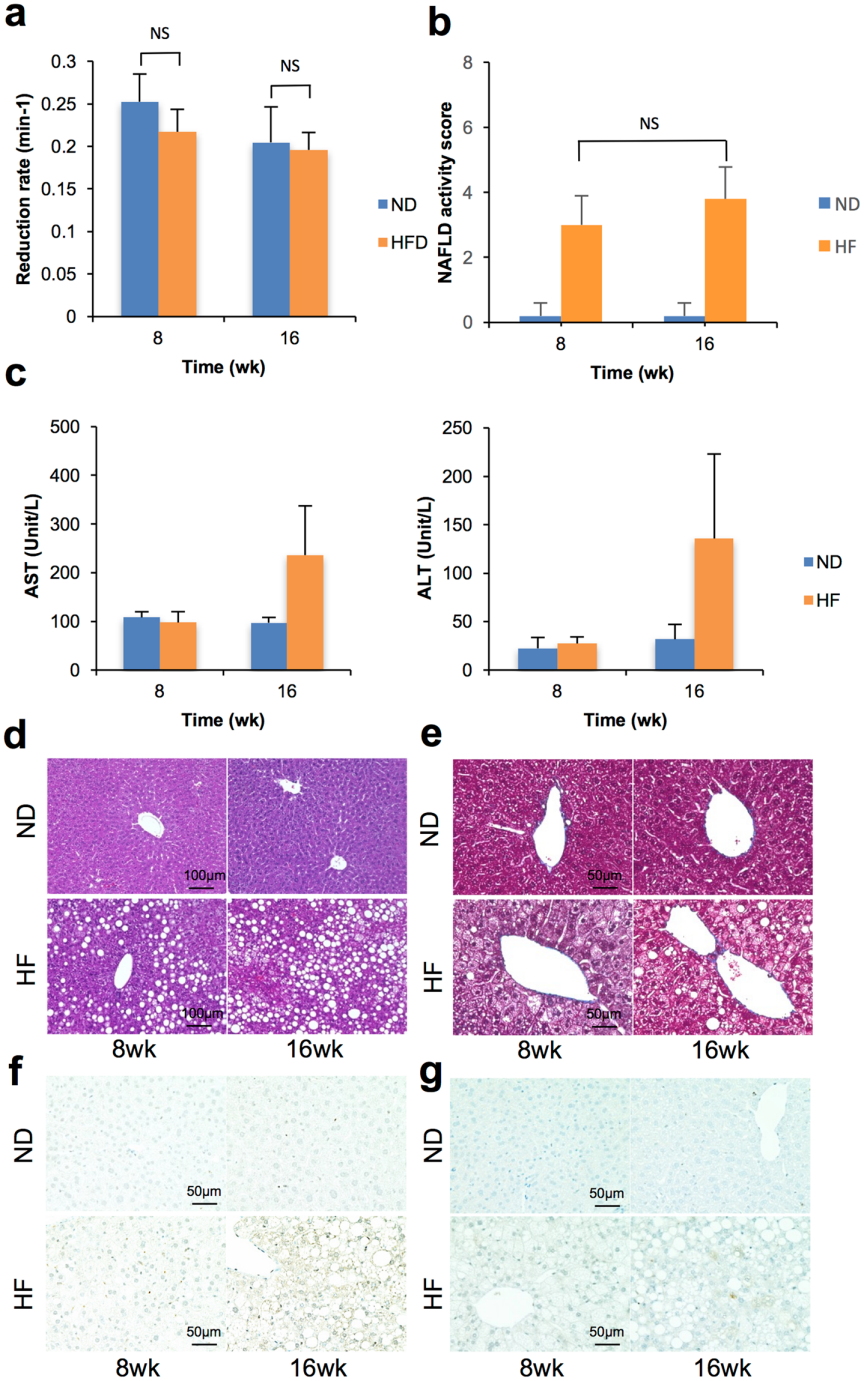
Redox status and pathological analysis after 8 and 16 weeks in HF diet mice (n = 5). (**a**) The reduction rate calculated by intensity change was dependent on the time course from 1 to 13 min after injection in control mice (blue bar) and dietary treated mice (yellow bar). (**b**) Plasma biochemistry for alanine transaminase (ALT) and aspartate transaminase (AST) after 8 and 16 weeks. (**c**) Histopathological findings with H/E staining. (**d**) Fibrosis assessment with Masson’s trichrome staining. (**e**) Immunohistochemistry of 8-OHdG for assessment of nuclei oxidized. (**f**) Immunohistochemistry of 4-HNE for assessment of lipid peroxidation.

Oxidative stress was assessed by immunohistochemical staining of 8-OHdG (Fig. 2a) and 4-HNE (Fig. 2b), which are indicative of nucleic oxidation and membrane lipid peroxidation, respectively. In MCD mice, nucleic oxidation was measured after 4 weeks and lipid peroxidation after 6 weeks. Cytosolic total antioxidant capacity (TAC) was significantly reduced in the MCD groups from an early phase compared with the control group (Fig. 2c). Moreover, the production of ROS was significantly increased at 4, 6 and 8 weeks in the MCD groups (Fig. 2d).

### Redox metabolic imaging of the mitochondrial phantom

**Figure 3:**
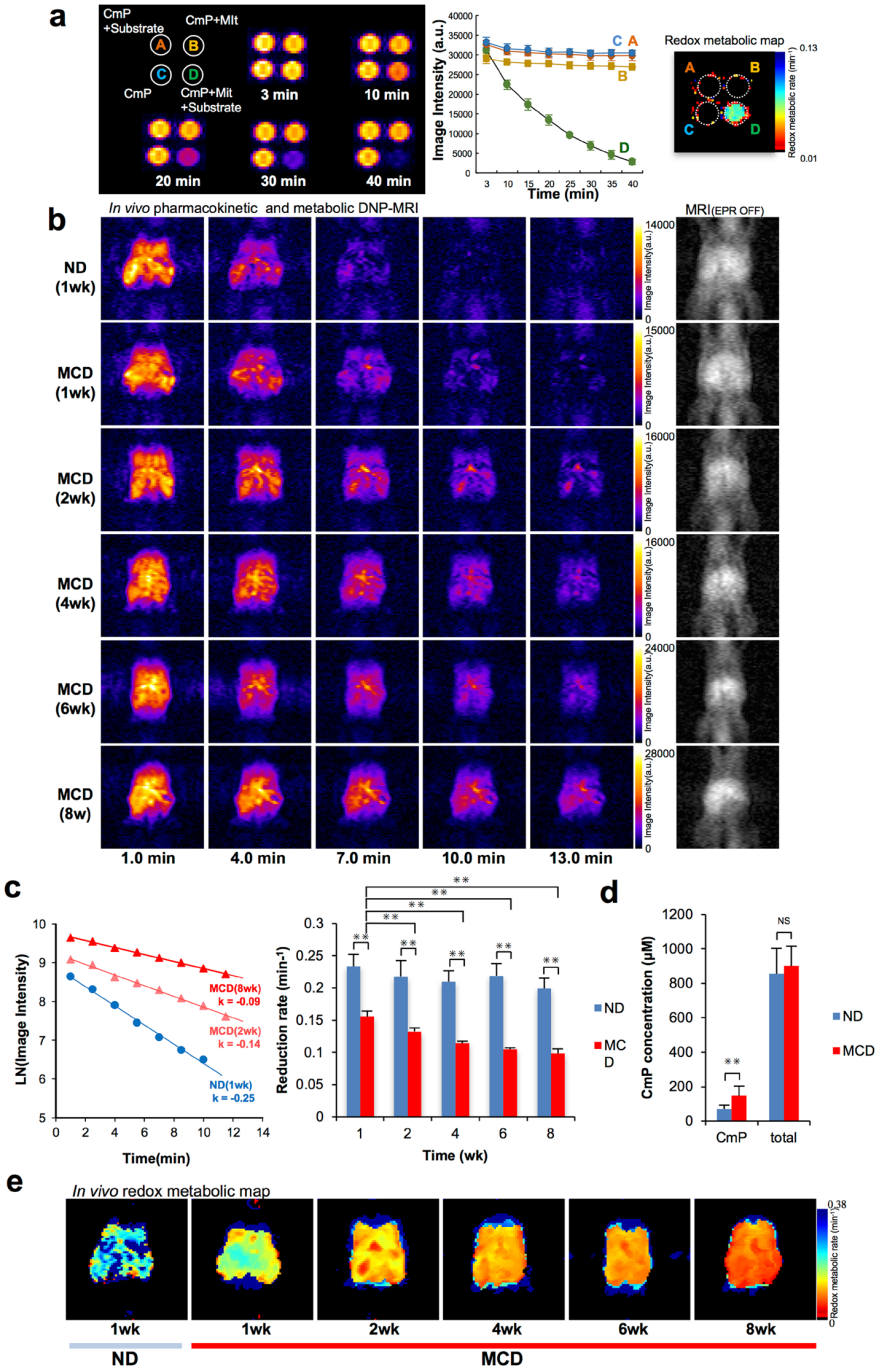
Redox metabolic imaging using *in vivo* DNP-MRI. (**a**) DNP-MRI of mitochondrial redox phantom using CmP as a redox probe. Tube A, 0.3 mM CmP and substrates (20 mM succinate and 20 mM NADH). Tube B, 0.3 mM CmP and 2 mg/mL mitochondria. Tube C, 0.3 mM CmP. Tube D, 0.3 mM CmP, 2 mg/mL mitochondria and substrates (20 mM succinate and 20 mM NADH. Image intensity in each tube was plotted. Each pixel in the redox metabolic map represents the reduction rate of CmP. (**b**) Temporal changes in DNP-MRI at 1, 2, 4, 6 and 8 weeks after intravenous injection of CmP in MCD mice (n = 5, each group). (**c**) The reduction rate, calculated by the change in intensity, was dependent on the time course from 1 to 13 min after injection in control mice (blue bar) and dietary-treated mice (red bar). Results represent the mean ± SD (n = 5 per group). ^✳✳^p < 0.01. The reduction rate was confirmed correlation with NAS (Spearman’s p = −0.8610). (**d**) Analysis of the oxidized form of CmP and total (oxidized and reduced form) of CmP in liver. The probe intensity was measured by ESR X-band with homogenates of liver tissue after 2 weeks in MCD mice (n = 5). Total CmP measured after re-oxidative treatment with ferricyanide. Results represent the mean ± SD (n = 5 per group). ^✳✳^p < 0.01. (**e**) *In vivo* redox metabolic mapping of nitroxide CmP in control mice and after 1, 2, 4, 6 and 8 weeks. The reduction rate of CmP were evaluated at each pixel and mapped on MRI.

Phantom experiments were carried out to examine the capability of CmP to participate in mitochondrial redox reaction and demonstrated the time dependent changes in image contrast using isolated mitochondria (Mit) and their substrates (succinate, ADP and NADH). Figure 3a shows the schematics of four phantom tubes and their DNP-MRI. As expected, all tubes showed significant enhancement and image intensity did not change without mitochondria (tube A) or substrates (tube B). However, tube D, which contained mitochondria and substrates, a time-dependent loss in image intensity was noticed. This result suggested that mitochondrial redox metabolism in electron transfer chain could be monitored by DNP-MRI and CmP as a redox probe.

### In vivo redox metabolic imaging in MCD mice

To monitor liver redox status non-invasively, *in vivo* DNP-MRI was performed after 1, 2, 4, 6 and 8 weeks of MCD dietary treatment in living mice. We also monitored same number of mice fed with a normal diet as control groups (n = 5 per group). CmP, a nitroxyl radical, was used as the redox imaging probe in this study. Enhanced DNP-MRI was conducted every 90 s from 1 min to 13 min after intravenous injection of CmP. The distribution of the enhanced signal area was observed in the whole liver at 1 min after injection (Fig. 3b). The intensity of the first image after CmP injection was increased, according to the duration of feeding. The intensity of DNP-MRI signals decreased during each experiment. For quantitative assessment, the reduction rate of DNP signal enhancement was calculated using pharmacokinetic DNP-MRI (Fig. 3c). Interestingly, even 1 week after starting MCD treatment without histopathological change, the reduction rate was significantly decreased, with further decreases after longer feeding intervals. In the control group, the reduction rate of CmP in liver was stable in all groups for each feeding period. The reduction rate was confirmed a correlation with NAS. Because the membrane permeability of the radical probe is altered by symptoms of disease progression, including inflammation, which was considered likely to affect the results, the oxidized form and total CmP (sum of the reduced and oxidized form) in the liver were measured quantitatively by X-band EPR to confirm that the findings reflected the redox reaction of the radical probe (Fig. 3d). The oxidized form of CmP was significantly decreased in liver homogenates from controls compared with the 2-week MCD group. Total CmP, which was measured after re-oxidative treatment with potassium ferricyanide, was not significantly different between control and MCD liver homogenates. These results suggest that the reduction rate monitored by *in vivo* DNP-MRI showed, not the difference of liver uptake and excretion, but radical loss of CmP by redox reaction. The redox maps constructed by calculation of the reduction rate in individual mice demonstrated that local redox alterations were dependent on the duration of the MCD diet.

### HF diet group

The HF diet group was assessed in the same manner as the MCD diet group to distinguish from simple steatosis. The reduction rate in the HF diet groups was not significantly different at either 8 or 16 weeks (Fig. 4a). Same as the reduction rate, assessment of histopathology with NAS system did not presented significant statistical change in HF mice (Fig. 4b). Despite the presence of lipid accumulation at 8 weeks, ALT and AST in plasma biochemistry were elevated after 16 weeks (Fig. 4c). In histopathological findings, hepatic steatosis was also observed in HF diet groups (Fig. 4d). Fibrosis and markers of oxidative stress were not increased compared with the MCD diet groups (Fig. 4e–g).

### Assessment of redox dynamics of CmP in liver tissue

**Figure 5:**
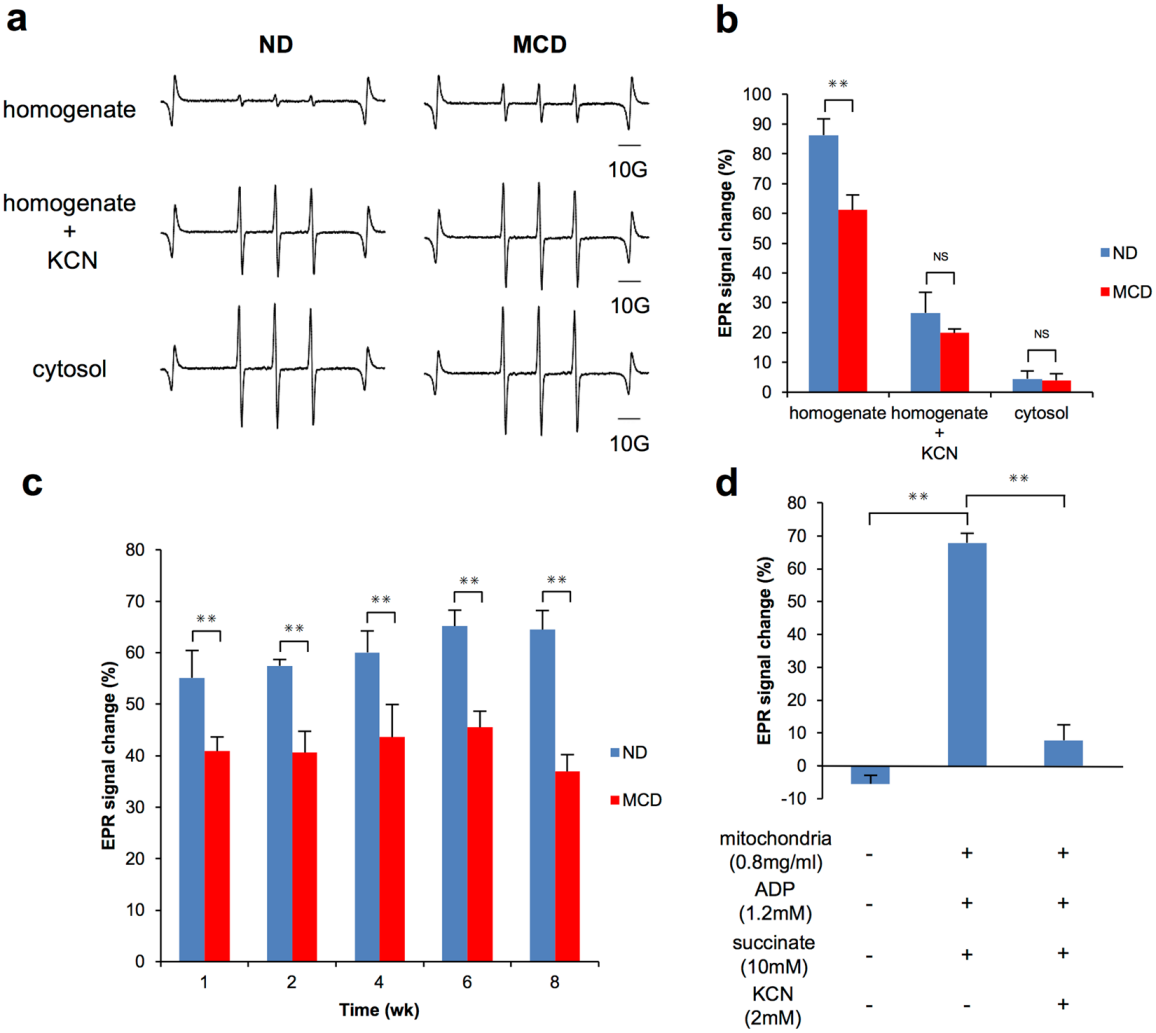
Assessment of CmP dynamics in liver tissue. (**a**) A typical ESR signal attenuation for 5 min of CmP in 5-fold diluted liver tissue homogenate solution. The sample diluted by homogenate buffer contained 70 mM sucrose, 220 mM mannitol, 0.2% BSA, 2 mM HEPES, 1 mM EGTA, 10 mM MgCl_2_ and 20 mM KH_2_PO_4_, pH 7.2. (**b**) Samples obtained after 2 weeks from the MCD mice group and the control group (n = 5). The CmP reduction rate was measured in liver tissue homogenates by ESR X-band and the relationship between mitochondria and CmP reactions was confirmed. After the addition of KCN, the CmP reduction rate was measured in the same manner. The reaction between CmP and cytosol was also measured in the same way. Results represent the mean ± SD (n = 5 per group). ^✳✳^p < 0.01. (**c**) Sample obtained after 1, 2, 4, 6 and 8 weeks in MCD mice and control groups (n = 5). The reduction rate of CmP was assessed in mitochondrial fractions (400 μg protein) in the presence of rotenone (1 mM), NADH (1 nM), ADP (1.2 mM) and succinate (10 mM). Results represent the mean ± SD (n = 5 per group). ^✳✳^p < 0.01. (**d**) Samples obtained after 8 weeks in mice fed a normal diet. The mitochondrial fraction isolated from liver tissue (400 μg protein) was measured by the reduction rate of CmP by ESR X-band under several conditions. Results represent the mean ± SD (n = 5 per group). ^✳✳^p < 0.01.

To clarify the mechanisms, the redox reaction between CmP and liver homogenates was directly monitored by X-band EPR. The EPR signal of CmP was reduced by freshly prepared liver homogenates. The EPR signal change at 5 min was significantly lower in the MCD groups than the control groups (Fig. 5a,b). Interestingly, these differences were completely inhibited by the addition of potassium cyanide (KCN), an inhibitor of complex IV in the mitochondrial electron transfer chain (ETC). Furthermore, CmP was not reduced by cytosol in either group (Fig. 5a,b). Because the difference between the control and NASH group in CmP redox metabolism was abolished by inhibition of the mitochondrial ETC in liver homogenate solutions, we next confirmed the mitochondrial metabolism of Cmp using the direct reaction between CmP and isolated mitochondria prepared from different stages of NASH. In the case of direct reaction of mitochondria, the redox metabolism of CmP (ratio of CmP reduction) revealed significant differences between the MCD and the control group in each feeding period, and these results corresponded with the results of *in vivo* DNP-MRI experiments (Fig. 5c). Furthermore, to confirm the mitochondrial-dependent redox metabolism of CmP, the reaction between CmP and mitochondria was assessed in detail. The reaction between isolated mitochondria from control mice (n = 3) and CmP was verified in the presence of stimulators (succinate, ADP) and an inhibitor (KCN). Figure 5d shows the rate of change of CmP for 30 min. Without mitochondria, there was almost no change in CmP, whereas 70% of CmP was reduced by the addition of mitochondria and succinate or ADP. Furthermore, KCN treatment suppressed 90% of the CmP reduction. Additional studies were performed to confirm the redox reaction between mitochondria and CmP by changing the conditions of the mixed solution added to CmP (Supplementary Fig. S2). The addition of mitochondria was a prerequisite for the metabolism of CmP. Metabolism of CmP was accelerated when the addition of substances associated with mitochondrial ETC and the redox reaction of CmP was inhibited without nicotinamide adenine dinucleotide (NADH).

## Discussion

With the global rise in the prevalence of NAFLD/NASH^1,40^, several non-invasive diagnostic methods have been explored in recent years^6^. However, due to their lack of sensitivity and specificity, currently available non-invasive tests for NASH have limited use, and the development of a reliable, non-invasive diagnostic method for NASH is desirable. Changes in diet have led to NASH becoming a major problem in children, with the incidence of NAFLD ranging from 2.6% to 9.6% in the general paediatric population^40,41^, rising to between 22.5% and 44.0% in obese children^40,42^. Paediatric NAFLD patients require long-term observation to identify disease progression to NASH. Furthermore, a non-invasive diagnostic strategy is strongly desirable for the paediatric population because liver biopsy is associated with pain and a higher risk of complications than in adult patients.

This is the first study to directly investigate the liver redox status related mitochondrial function of a NASH mouse model using non-invasive, *in vivo* DNP-MRI and CmP as radical probe unlike the previous reports aiming for clinical application of DNP-MRI. The reduction rate, as estimated from the enhanced DNP signal, was significantly altered at 1, 2, 4, 6 and 8 weeks in all MCD mice, accompanied by disease progression with statistical analysis of correlation. In addition, a direct reaction between liver homogenates and CmP strongly supported the results of *in vivo* DNP-MRI, and the dynamics of CmP reduction changes corresponded to disease progression, with excess oxidative stress detected earlier than by immunohistochemical analysis of 8-OHdG and 4-HNE. Disease progression of NASH was visualized as a redox map (Fig. 3e), enabling quantitative assessment at an early stage of NASH by *in vivo* imaging. In general, NASH-like histopathological changes are observed in the liver from 6 to 8 weeks in the MCD diet mouse model. Although the HF diet groups also had elevated AST and ALT levels in serum biochemistry, evaluation of NAFLD patients should be based on metabolic risk factors, not on these biomarkers^43^. In this study, signs of progression to NASH in all measured mice were detected by mitochondrial metabolic imaging with *in vivo* DNP-MRI before histopathological change. Previous reports suggested that the oxidative stress is responsible for disease progression^3,10–12^. Therefore, our results suggest that mitochondrial metabolism and antioxidant homeostasis effected from oxidative stress in NASH were responsible for triggering disease progression, and these metabolic changes were identified at an early phase of the change in redox status using *in vivo* DNP-MRI. One of the primary concerns in clinical practice is to establish a diagnostic method that differentiates between NASH and liver steatosis. In contrast to MCD mice, the CmP reduction in HF mice was not significantly altered on DNP-MRI, which may be explained by the HF diet inducing only a low level of oxidative stress that does not influence disease progression. *In vivo* redox status may be a valuable diagnostic biomarker for distinguishing between simple liver steatosis and NASH. Although we previously reported that the redox alteration on liver cirrhosis mice model by repeated administration of dimethylnitrosamine (DMN)^44^ was observed, it showed the reduction change after destruction of liver cells. In DMN induced liver cirrhosis model, the total CmP content was significantly increased in the liver region due to decreased clearance of CmP by strong hepatocellular injury. On the other hand, present experiment showed that the reduction rate of CmP was decreased at early stage of MCD treatment (1–2 week) before destruction of liver cells, because the clearance of CmP, mitochondrial oxygen consumption and histological observation of liver cells (only steatosis was observed) were normal.

KCN, a mitochondrial ETC inhibitor, suppressed the difference in CmP reduction between the MCD group and the control group, and cytosol was not related to CmP redox reactions. CmP reduction is strongly related to the mitochondrial ETC (Fig. S1). We thought that mitochondrial redox metabolism, especially the ETC, is a key factor that influences *in vivo* CmP reduction in MCD mice. Several previous reports have also suggested that the pathology of NASH is linked to mitochondrial abnormalities^15,45^. Cytochrome c oxidase of mitochondrial complex IV is inhibited by lipid peroxidation in NASH^13^, and the relationship between the failure of mitochondrial ETC and the redox reaction of CmP was implied. In addition, direct reaction between CmP and isolated mitochondria from the MCD mice showed significant differences, similar to *in vivo* DNP-MRI, and these data were indicative of functional changes in the metabolism of the mitochondrial ETC during the early phase of NASH progression. The relationship between CmP and mitochondrial ETC was confirmed under different conditions, including stimulators (succinate and ADP) and an inhibitor. These results showed that mitochondrial function, especially the ETC, is strictly related to the *in vivo* reduction of CmP^46^.

Past studies reported that CmP is oxidized by ROS to oxonium cation, and then reduced to hydroxylamine by reductants such as glutathione and NADH. Because hydroxylamine is non-magnetic, it has no signal in DNP-MRI. It is possible to visualize the biological redox status of tissue by monitoring oxidization and reduction reactions that depend on the *in vivo* environment of the nitroxyl radical in magnetic resonance methods^47^. A recent study suggested that the redox reaction of CmP was related to environmental changes in the liver tissue of NASH, which was affected by mitochondrial ETC dysfunction and reduced antioxidant species.

Over the next two decades, it is expected that cirrhosis and end-stage liver disease related to NASH will become the leading indications for liver transplantation^3,48^. Despite the development of several non-invasive diagnostic methods for NASH, unfortunately, technology to precisely diagnose NASH has been lacking. The most important findings of this study were that mitochondrial redox alterations were confirmed with NASH progression *in vivo*, and precise and non-invasive detection of NASH liver by monitoring *in vivo* mitochondrial redox reactions with DNP-MRI could be achieved. Our result also demonstrated that mitochondrial redox metabolism assessed *in vivo* is a key diagnostic factor for NASH, and it may enable NASH to be distinguished from NAFLD patients in clinical practice. Moreover, this technique has potential value for diagnostic imaging techniques and clinical follow-up for NASH patients.

## Materials and Methods

### Chemicals

3-carbamoyl-2,2,5,5-tetramethyl-1-pyrrolidine-1-oxyl (carbamoyl-PROXYL) was purchased from Aldrich Chemical Co. (Milwaukee, WI, USA). Rotenone was purchased from MP Biomedicals (Santa Ana, CA, USA). Succinate and KCN were purchased from Wako Pure Chemical Industries (Osaka, Japan). Adenosine 5′-diphosphate sodium salt (ADP) was purchased from Sigma-Aldrich (St Louis, MO, USA). 8-OHdG was purchased from Bioss Antibodies (Woburn, MA, USA). 4-hydroxy-2-neonal monoclonal antibody was purchased from JaICA (Shiduoka, Japan). All other chemicals were commercially available, and of reagent grade quality.

### Animals and dietary treatments

All animal care and experiment protocols were approved by the Animal Ethics Committee of Kyushu University Faculty of Medicine and were carried out according to the recommendations of the Committee for Care and Use of Laboratory Animals, Kyushu University Faculty of Medicine.

### MCD diet treatment

Male C57BL/6 mice, aged 5 weeks, were purchased from Charles River Laboratories Japan, Inc. (Yokohama, Japan), and maintained in temperature- and light-controlled chambers (24 °C, 12-h/12-h light-dark cycle). An acute pathological model was developed by feeding the mice with a methionine-choline-deficient (MCD) diet (Oriental Yeast Co., Tokyo, Japan). Prior to experiments, all animals were fed a normal diet for 1 week for acclimation, and had free access to tap water and appropriate food (MF diet, Oriental Yeast Co., Tokyo, Japan). At the age of 6 weeks, they were divided into 10 groups (n = 5), and studied after 1, 2, 4, 6 and 8 weeks on the MCD diet or a normal diet.

### HF diet treatment

C57BL/6 mice, aged 9 weeks, were purchased from Charles River Laboratories Japan, Inc. (Yokohama, Japan), and maintained in temperature- and light-controlled chambers (24 °C, 12-h/12-h light-dark cycle). A HF model was developed by feeding the mice a HF diet containing 60% of total calories from fat (Oriental Yeast Co., Tokyo, Japan). During the acclimatization period for 1 week prior to experiments, all animals received a normal diet with free access to tap water and appropriate food (MF diet, Oriental Yeast Co., Tokyo, Japan). At the age of 10 weeks, they were divided into 4 groups (n = 5), and studied after 8 and 16 weeks on the HF diet or a normal diet.

### Histopathology

Liver tissues were fixed in 10% formalin, cut into 5-μm sections, and stained with haematoxylin and eosin (H&E). Hepatic fibrosis was evaluated using Masson’s trichrome stain according to the manufacturer’s instructions. Assessment of disease progression was assessed by the NAFLD activity score (NAS) as described by Kleiner *et al*.,^49^ with separate scores for steatosis (0–3), hepatocellular ballooning (0–2), and lobular inflammation (0–3). The NAS is the sum of these scores, and values of 5 are reported to be correlated with a diagnosis of NASH. Liver sections were routinely deparaffinized and immunostained for 4-HNE and 8-OHdG, as reliable markers of lipid peroxidation^50^ and oxidative DNA damage^51,52^, respectively. For 8-OHdG staining, the sections were microwaved in 10 mM citrate buffer (pH 6.0) for 15 min for antigen retrieval. After three washes with phosphate-buffered saline (PBS), the sections were sequentially treated with 3% skimmed milk in PBS for 30 min at room temperature to block non-specific binding, 1 μg/mL of anti-8-OHdG polyclonal antibodies or 4-HNE monoclonal antibodies, and a peroxidase-avidin complex as the secondary antibody (EnvVsion + kits; Dako Japan Co. Ltd, Kyoto, Japan). The sections were observed under a microscope (BioRevo BZ-9000; Keyence, Osaka, Japan).

### Plasma biochemistry

Male C57/BL6 mice on the MCD diet were anesthetized with 2% isoflurane after different time periods. Blood samples were collected from the right atrium via cardiac puncture for measurement of ALT and AST. The blood samples were immediately centrifuged at 1,000 × g for 30 min at 4 °C, and the plasma was aliquoted and stored at −80 °C. Plasma ALT and AST levels were measured with the Fuji DRI-CHEM NX500V system (Fuji Film, Tokyo, Japan).

### Evaluation of total antioxidant capacity

The total antioxidant capacity of liver tissue was evaluated with a colorimetric OxiSelect Total Antioxidant Capacity Assay Kit (Cell Biolabs Inc., San Diego, CA, USA). Liver tissues were homogenized with the same volume of PBS. The homogenate was centrifuged at 10,000 × g for 15 min at 4 °C. The supernatant was collected and assays were performed according to the standard protocols.

### Measurement of mitochondrial superoxide anion production

Freshly prepared isolated mitochondria were treated with 5 μM MitoSOX-red (Thermo Fisher Scientific Inc.) for 30 min at 37 °C and fluorescence was measured according to the standard protocols.

### Mitochondrial redox phantom imaging by DNP-MRI

Mitochondrial redox phantom imaging was performed with a low magnetic field, *in vivo* DNP-MRI system (Keller-Japan redox Inc.). The external magnetic field (B_0_) for EPR irradiation and MRI was fixed at 15 mT, and the radiofrequencies for EPR irradiation and MRI were 455 MHz and 683 kHz, respectively. Four phantom tubes were prepared as shown in Fig. 3a. Tube A contained 0.3 mM CmP and substrates (20 mM succinate and 20 mM NADH). Tube B contained 0.3 mM CmP and 2 mg/mL mitochondria. Tube C contained 0.3 mM CmP. Tube D contained 0.3 mM CmP, 2 mg/mL mitochondria and substrates (20 mM succinate and 20 mM NADH. These tubes were horizontally set in the resonator. DNP-MRI measurement was immediately started and continuously measured for up to 40 minutes. The experiments were repeated three times with freshly prepared solutions. Phantom DNP-MRI images were obtained at 3, 5, 10, 15, 20, 25, 30, 35 and 40 min after starting measurement. The redox map was obtained by the slope of the enhanced DNP image intensity of each pixel from four pharmacokinetic images using a custom Excel macro program. The scanning conditions for the DNP-MRI experiment were as follows: power of EPR irradiation, 12 W; flip angle, 90°; repetition time (T_R_) × echo time (T_E_) × EPR irradiation time (TEPR), 500 × 25 × 250 ms; number of accumulation, 2; slice thickness, 100 mm including the whole thickness of the mouse; phase-encoding steps, 32; field of view (FOV), 40 × 40 mm; and matrix size, 64 × 64 after reconstruction. The DNP-MRI data were analysed using ImageJ software^53^.

### Redox imaging by *in vivo* DNP-MRI

In vivo redox metabolic imaging was performed with a low magnetic field, *in vivo* DNP-MRI system. A rectangular, one-turned curved surface coil (longitudinal 20 mm, lateral 32 mm) was constructed for EPR irradiation during liver imaging in this study^54^. Mice were anesthetized with 2% isoflurane and then secured in a dorsal recumbent position on a special holder with adhesive skin tape. During the procedure, the body temperature of the mice was kept at 37 ± 1 °C with a heating pad. The animal holder was placed in centre of the resonator and *in vivo* DNP-MRI scanning of the upper abdomen was started immediately after intravenous injection of carbamoyl-PROXYL (150 mM CmP in half saline, 10 μL/g body weight). Pharmacokinetic DNP-MRI images were obtained at 1, 2.5, 4, 5.5, 7, 8.5, 10, 11.5 and 13 min after administration. Normal MRI images with 1 and 10 accumulations were obtained without EPR irradiation. The *in vivo* redox map was obtained by the slope of the enhanced DNP image intensity of each pixel from four pharmacokinetic images using a custom Excel macro program. The scanning conditions for the *in vivo* DNP-MRI experiment were as follows: power of EPR irradiation, 7 W; flip angle, 90°; repetition time (T_R_) × echo time (T_E_) × EPR irradiation time (TEPR), 500 × 25 × 250 ms; number of accumulation, 2; slice thickness, 100 mm including the whole thickness of the mouse; phase-encoding steps, 32; field of view (FOV), 40 × 40 mm; and matrix size, 64 × 64 after reconstruction. The *in vivo* DNP-MRI data were analysed using ImageJ software^53^.

### Quantitative analysis of oxidized and total CmP in liver

Carbamoyl-PROXYL (150 mM, 10 μl/g body weight) solution was intravenously injected into mice fed a normal control diet (n = 5) or an MCD diet (n = 5) for 2 weeks. Mice were sacrificed for collection of liver tissue at 5 min after probe injection. Fresh liver tissues were homogenized with half saline using a Potter-Elvehjem grinder on ice. The nitroxyl radical concentration of each sample was measured by X-band EPR. Total carbamoyl-PROXYL was measured after the addition of ferricyanide. Measurement parameters for X-band EPR were as follows: microwave frequency, 1.0 GHz; microwave power, 1.0 mW; centre of field, 37.8 mT; modulation width, 0.03 mT; sweep time, 30 s; sweep width, 5.0 mT; and time constant, 0.03 s.

### Assessment of CmP redox metabolism in liver tissue using X-band EPR

After 2 weeks’ feeding, 3 mice on the MCD diet and 3 mice on the normal control diet were sacrificed. Homogenate solution was made by homogenate buffer containing 70 mM sucrose, 220 mM mannitol, 0.2% BSA, 2 mM HEPES, 1 mM EGTA, 10 mM MgCl_2_ and 20 mM KH_2_PO_4_, pH 7.2. The reduction rate of Cmp radicals was measured by X-band EPR at 5 min after mixing of CmP (20 μM) and liver homogenates (×5). Under the condition of adding KCN and cytosol, CmP radical concentrates were monitored by X-band EPR. The reduction rate was calculated using the peak height of CmP radical spectra at 30 min later. Measurement parameters of X-band EPR were as follows: microwave frequency, 1 GHz; microwave power, 10 mW; centre of field, 328.2 mT; modulation width, 5.0 mT; sweep time, 60 s; sweep width, 5.0 mT; and time constant, 0.03 s.

### Mitochondrial isolation

Liver mitochondria were prepared by differential centrifugation of liver homogenates^55^ using an ice-cold mitochondria isolation buffer containing 70 mM sucrose, 220 mM mannitol, 0.2% BSA, 10 mM KH_2_PO_4_, 2 mM HEPES and 1 mM EGTA, pH 7.2. Liver tissue homogenates were centrifuged at 600 × g for 10 min at 4 °C. The supernatant was decanted and stored. The remaining pellets contain tissue fragments and some mitochondria. Protease inhibitors were added to the isolation buffer to prevent protein degradation. The stored supernatants were centrifuged at 8,000 × g for 10 min at 4 °C. The resultant supernatant was discarded along with any lightly packed, pink microsomes. Lightly packed tan mitochondria were retained. The pellets were suspended with mitochondria isolation buffer (5 ml) and centrifuged at 8,000 × g for 10 min at 4 °C. The final pellets were weighed after discarding the supernatant. Based on the results of protein assays, the mitochondrial final concentration was adjusted to 0.8 mg/ml.

### CmP redox metabolism in isolated mitochondria

The reduction rate of CmP radical probes was measured by X-band EPR at 30 min after adding 5 μM CmP, 1 μM Rotenone, 0.8 mg/ml mitochondria, 1 mM NADH, 1.2 mM ADP and 10 mM succinate. Measurement parameter for X-band EPR were as follows: microwave frequency, 1 GHz; microwave power, 10 mW; centre of field, 328.2 mT; modulation width, 5 mT; sweep time, 60 s; sweep width, 5.0 mT; and time constant, 0.03 s.

### Statistical analysis

All data are presented as the means ± standard deviations and differences between mean values were evaluated by One way ANOVA with post hoc Tukey-kramer HSD tests. p < 0.05 was considered to denote a statistically significant difference. Spearman rank correlation analysis was used to estimate the relationship between the reduction rate and NAS. All statistical analyses were performed using JMP^®^ 13 (SAS Institute Inc., Cary, NC, USA).

## Electronic supplementary material

Supplementary Information

## References

[1] Angulo P (2002). Nonalcoholic fatty liver disease. N Engl J Med.

[2] Bhala N (2011). The natural history of nonalcoholic fatty liver disease with advanced fibrosis or cirrhosis: an international collaborative study. Hepatology.

[3] Musso G, Cassader M, Gambino R (2016). Non-alcoholic steatohepatitis: emerging molecular targets and therapeutic strategies. Nat Rev Drug Discov.

[4] Bugianesi E (2002). Expanding the natural history of nonalcoholic steatohepatitis: from cryptogenic cirrhosis to hepatocellular carcinoma. Gastroenterology.

[5] Schwenzer NF (2009). Non-invasive assessment and quantification of liver steatosis by ultrasound, computed tomography and magnetic resonance. J Hepatol.

[6] Wieckowska A, McCullough AJ, Feldstein AE (2007). Noninvasive diagnosis and monitoring of nonalcoholic steatohepatitis: present and future. Hepatology.

[7] Janiec DJ, Jacobson ER, Freeth A, Spaulding L, Blaszyk H (2005). Histologic variation of grade and stage of non-alcoholic fatty liver disease in liver biopsies. Obes Surg.

[8] Younossi ZM (1998). Nonalcoholic fatty liver disease: assessment of variability in pathologic interpretations. Mod Pathol.

[9] Gawrieh S, Knoedler DM, Saeian K, Wallace JR, Komorowski RA (2011). Effects of interventions on intra- and interobserver agreement on interpretation of nonalcoholic fatty liver disease histology. Ann Diagn Pathol.

[10] Hebbard L, George J (2011). Animal models of nonalcoholic fatty liver disease. Nat Rev Gastroenterol Hepatol.

[11] Jorgacevic B (2014). Dynamics of oxidative/nitrosative stress in mice with methionine-choline-deficient diet-induced nonalcoholic fatty liver disease. Hum Exp Toxicol.

[12] Marra F, Gastaldelli A, Svegliati Baroni G, Tell G, Tiribelli C (2008). Molecular basis and mechanisms of progression of non-alcoholic steatohepatitis. Trends Mol Med.

[13] Paradies G, Ruggiero FM, Gadaleta MN, Quagliariello E (1992). The effect of aging and acetyl-L-carnitine on the activity of the phosphate carrier and on the phospholipid composition in rat heart mitochondria. Biochim Biophys Acta.

[14] Begriche K, Igoudjil A, Pessayre D, Fromenty B (2006). Mitochondrial dysfunction in NASH: causes, consequences and possible means to prevent it. Mitochondrion.

[15] Caldwell SH (1999). Mitochondrial abnormalities in non-alcoholic steatohepatitis. J Hepatol.

[16] Murphy MP (2009). How mitochondria produce reactive oxygen species. Biochem J.

[17] Skulachev VP (1996). Role of uncoupled and non-coupled oxidations in maintenance of safely low levels of oxygen and its one-electron reductants. Q Rev Biophys.

[18] Lambert AJ, Brand MD (2004). Inhibitors of the quinone-binding site allow rapid superoxide production from mitochondrial NADH:ubiquinone oxidoreductase (complex I). J Biol Chem.

[19] Lurie DJ, Lawrence DMB, Bell H, Mallard JR (1988). Proton-Electron Double Magnetic Resonance Imaging of Free Radical Solutions. Jornal of Magnetic Resonance.

[20] Hyodo F, Ito S, Yasukawa K, Kobayashi R, Utsumi H (2014). Simultaneous and spectroscopic redox molecular imaging of multiple free radical intermediates using dynamic nuclear polarization-magnetic resonance imaging. Anal Chem.

[21] Overhauser AW (1953). Polarization of Nuclei inMetals. Physical Review.

[22] Kuppusamy P (2002). Noninvasive imaging of tumor redox status and its modification by tissue glutathione levels. Cancer Res.

[23] Hu G, Lyeth BG, Zhao X, Mitchell JB, Watson JC (2003). Neuroprotection by the stable nitroxide 3-carbamoyl-proxyl during reperfusion in a rat model of transient focal ischemia. J Neurosurg.

[24] Hyodo F, Matsumoto K, Matsumoto A, Mitchell JB, Krishna MC (2006). Probing the intracellular redox status of tumors with magnetic resonance imaging and redox-sensitive contrast agents. Cancer Res.

[25] Soule BP (2007). The chemistry and biology of nitroxide compounds. Free Radic Biol Med.

[26] Canney PA, Dean S (1990). Transforming growth factor beta: a promotor of late connective tissue injury following radiotherapy?. Br J Radiol.

[27] Couet WR, Brasch RC, Sosnovsky G, Tozer TN (1985). Factors affecting nitroxide reduction in ascorbate solution and tissue homogenates. Magn Reson Imaging.

[28] Swartz HM (1990). Principles of the metabolism of nitroxides and their implications for spin trapping. Free Radic Res Commun.

[29] Wilcox CS (2010). Effects of tempol and redox-cycling nitroxides in models of oxidative stress. Pharmacol Ther.

[30] Eto H (2015). Redox imaging of skeletal muscle using in vivo DNP-MRI and its application to an animal model of local inflammation. Free Radic Biol Med.

[31] Lurie DJ, Davies GR, Foster MA, Hutchison JM (2005). Field-cycled PEDRI imaging of free radicals with detection at 450 mT. Magn Reson Imaging.

[32] Utsumi H (2006). Simultaneous molecular imaging of redox reactions monitored by Overhauser-enhanced MRI with 14N- and 15N-labeled nitroxyl radicals. Proc Natl Acad Sci USA.

[33] Krishna MC (2002). Overhauser enhanced magnetic resonance imaging for tumor oximetry: coregistration of tumor anatomy and tissue oxygen concentration. Proc Natl Acad Sci USA.

[34] Ahn KH, Scott G, Stang P, Conolly S, Hristov D (2011). Multiparametric imaging of tumor oxygenation, redox status, and anatomical structure using Overhauser-enhanced MRI-prepolarized MRI system. Magn Reson Med.

[35] Samouilov A (2014). *In vivo* proton-electron double-resonance imaging of extracellular tumor pH using an advanced nitroxide probe. Anal Chem.

[36] Koonjoo N (2014). In vivo Overhauser-enhanced MRI of proteolytic activity. Contrast Media Mol Imaging.

[37] Quintanilha AT, Packer L (1977). Surface localization of sites of reduction of nitroxide spin-labeled molecules in mitochondria. Proc Natl Acad Sci USA.

[38] Chapman DA, Killian GJ, Gelerinter E, Jarrett MT (1985). Reduction of the spin-label TEMPONE by ubiquinol in the electron transport chain of intact rabbit spermatozoa. Biol Reprod.

[39] Romestaing C (2008). Mitochondrial adaptations to steatohepatitis induced by a methionine- and choline-deficient diet. Am J Physiol Endocrinol Metab.

[40] Schwimmer JB (2006). Prevalence of fatty liver in children and adolescents. Pediatrics.

[41] Strauss RS, Barlow SE, Dietz WH (2000). Prevalence of abnormal serum aminotransferase values in overweight and obese adolescents. J Pediatr.

[42] Sartorio A (2007). Predictors of non-alcoholic fatty liver disease in obese children. Eur J Clin Nutr.

[43] Fracanzani AL (2008). Risk of severe liver disease in nonalcoholic fatty liver disease with normal aminotransferase levels: a role for insulin resistance and diabetes. Hepatology.

[44] Kawano T (2016). Noninvasive mapping of the redox status of dimethylnitrosamine-induced hepatic fibrosis using *in vivo* dynamic nuclear polarization-magnetic resonance imaging. Sci Rep.

[45] Nassir F, Ibdah JA (2014). Role of mitochondria in nonalcoholic fatty liver disease. Int J Mol Sci.

[46] Alecci M, Lurie DJ, Nicholson I, Placidi G, Sotgiu A (1996). Young Investigator Award presentation at the 13th Annual Meeting of the ESMRMB, September 1996, Prague. A proton-electron double-resonance imaging apparatus with simultaneous multiple electron paramagnetic resonance irradiation at 10 mT. MAGMA.

[47] Matsumoto K (2006). High-resolution mapping of tumor redox status by magnetic resonance imaging using nitroxides as redox-sensitive contrast agents. Clin Cancer Res.

[48] Wong RJ (2015). Nonalcoholic steatohepatitis is the second leading etiology of liver disease among adults awaiting liver transplantation in the United States. Gastroenterology.

[49] Kleiner DE (2005). Design and validation of a histological scoring system for nonalcoholic fatty liver disease. Hepatology.

[50] Esterbauer H, Schaur RJ, Zollner H (1991). Chemistry and biochemistry of 4-hydroxynonenal, malonaldehyde and related aldehydes. Free Radic Biol Med.

[51] Shibutani S, Takeshita M, Grollman AP (1991). Insertion of specific bases during DNA synthesis past the oxidation-damaged base 8-oxodG. Nature.

[52] Kasai H (1997). Analysis of a form of oxidative DNA damage, 8-hydroxy-2′-deoxyguanosine, as a marker of cellular oxidative stress during carcinogenesis. Mutat Res.

[53] Schneider CA, Rasband WS, Eliceiri KW (2012). NIH Image to ImageJ: 25 years of image analysis. Nat Methods.

[54] Matsumoto S (2007). Advantageous application of a surface coil to EPR irradiation in overhauser-enhanced MRI. Magn Reson Med.

[55] Venkatraman A (2004). Modification of the mitochondrial proteome in response to the stress of ethanol-dependent hepatotoxicity. J Biol Chem.

