# Supplementary material for: In vivo redox metabolic imaging of mitochondria assesses disease progression in non-alcoholic steatohepatitis
Source: Sci Rep. 2017 Dec 7;7:17170. doi: 10.1038/s41598-017-17447-2 (PMC5719423; doi:10.1038/s41598-017-17447-2)
Supplement: Supplementary file 1 — Supplementary Information [file 41598_2017_17447_MOESM1_ESM.pdf]

In vivo redox metabolic imaging of mitochondria assesses disease progression in non-alcoholic steatohepatitis

Ryosuke Nakata[1], Fuminori Hyodo[2,3]*, Masaharu Murata[1,3,4,*], Hinako Eto[2], Tomoko Nakaji[3], Takahito Kawano[1,3], Sayoko Narahara[1,3], Keiji Yasukawa[5], Tomohiko Akahoshi[1], Morimasa Tomikawa[1] & Makoto Hashizume[1,3,4]

[1]Department of Advanced Medical Initiatives, Faculty of Medical Sciences, Kyushu University, 3-1-1 Maidashi, Higashi-ku, Fukuoka 812-8582, Japan. [2]Department of Frontier Science for Imaging, School of Medicine, Gifu University, 1-1 Yanagido Gifu 501-1194, Japan. [3]Innovation Center for Medical Redox Navigation and [4]Center for Advanced Medical Innovation, Kyushu University, 3-1-1 Maidashi, Higashi-ku, Fukuoka 812-8582, Japan. [5]Laboratory of Advanced Pharmacology, Daiichi University of Pharmacy, 22-1 Tamagawa-machi, Minami-ku, Fukuoka 815-0037, Japan *These authors contributed equally to this work. All correspondence should be addressed to F.H. (e-mail: hyodof@redoxnavi.med.kyushu-u.ac.jp)

Supplementary Materials:

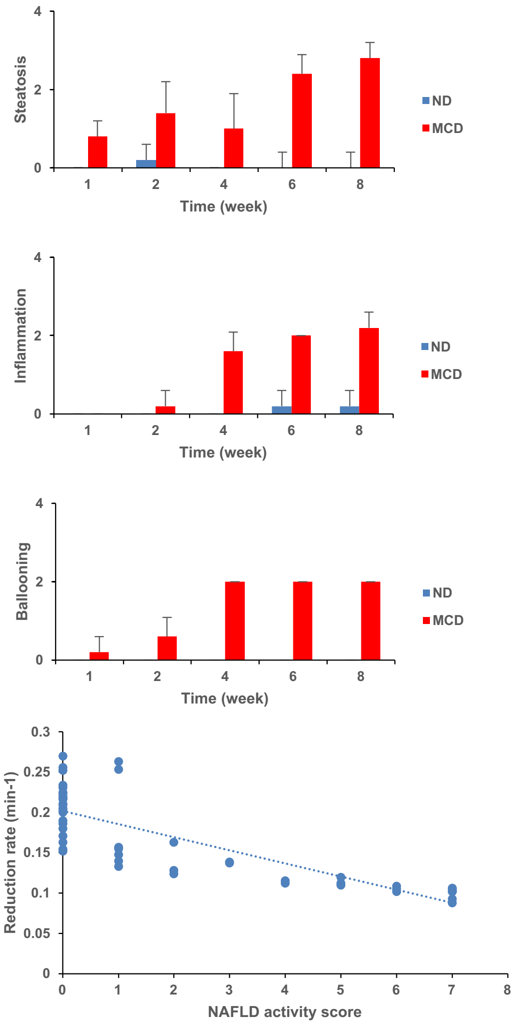

Supplement Figure S1

Assessment of disease progression was conducted with the NAFLD activity score with separate scores for steatosis (0-3), hepatocellular ballooning (0-2), and lobular inflammation (0-3). Spearman rank correlation analysis was used to estimate the relationship between the reduction rate and NAS. (Spearman's p=-0.8610)

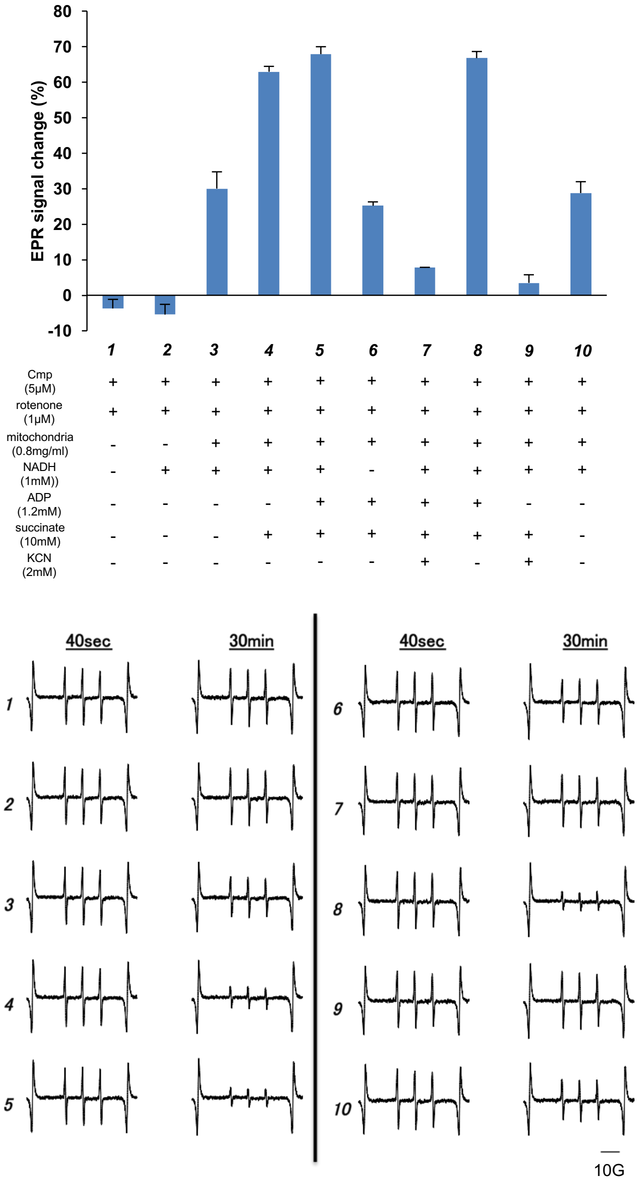

Supplement Figure S2

As an additional experiment, the relationship between CmP and mitochondria was verified. Mitochondrial dysfunction is associated with NASH, and mitochondria are involved in redox status homeostasis. Isolated mitochondria from control mice (n=3) were used to observe the CmP reaction after the application of substrates (succinate, ADP) and an inhibitor (KCN). The rate of change from

the oxidized to the reduced form over 30 min, without mitochondria, showed no changes in CmP form. After the addition of substrates, the reduced form of CmP was increased. The ETC breaks down after the addition of KCN and the CmP reaction is stopped. This suggests that the mitochondrial ETC may be linked to CmP reduction reactions.
